# Clinical significance of serum and mesangial galactose-deficient IgA1 in patients with IgA nephropathy

**DOI:** 10.1371/journal.pone.0206865

**Published:** 2018-11-02

**Authors:** Yukihiro Wada, Kei Matsumoto, Taihei Suzuki, Tomohiro Saito, Nobuhiro Kanazawa, Shohei Tachibana, Ken Iseri, Motonori Sugiyama, Masayuki Iyoda, Takanori Shibata

**Affiliations:** Division of Nephrology, Department of Medicine, Showa University School of Medicine, Tokyo, Japan; INSERM1163, FRANCE

## Abstract

**Introduction:**

Galactose-deficient IgA1 (Gd-IgA1) is a critical pathogenic factor for IgA nephropathy (IgAN), but its value as a disease-specific biomarker remains controversial. We aimed to clarify the clinical significance of Gd-IgA1 in patients with IgAN.

**Methods:**

We retrospectively reviewed 111 patients who were diagnosed with IgAN based on the findings of renal biopsies (RB) at Showa University Hospital since 2007. Serum Gd-IgA1 (s-Gd-IgA1) at the time of RB was compared among 111 IgAN patients, 18 Henoch-Schönlein purpura nephritis (HSPN) patients, 29 lupus nephritis (LN) patients, 28 ANCA-associated vasculitis (AAV) patients, and 13 minimal change disease (MCD) patients using ELISA with an anti-human Gd-IgA1-specific monoclonal antibody (KM55). We also immunohistochemically stained paraffin-embedded sections for mesangial Gd-IgA1 (m-Gd-IgA1) deposition using KM55.

**Results:**

Although levels of s-Gd-IgA1 were comparable among IgAN and HSPN, s-Gd-IgA1 levels were significantly elevated in patients with IgAN compared with LN, AAV and MCD (IgAN vs. HSPN, LN, AAV, and MCD: 16.2 ± 9.1 vs. 14.2 ± 10.8, p = 0.263; 12.7 ± 9.4, p = 0.008; 13.1 ± 7.3, p = 0.059; and 8.2 ± 4.8 μg/mL, p<0.001, respectively). Mesangial-Gd-IgA1 deposition was specifically detected in IgAN or HSPN. The increase in s-Gd-IgA1 significantly correlated with m-Gd-IgA1 positivity in patients with IgAN, and s-Gd-IgA1 elevation and m-Gd-IgA1 deposition were evident in patients with histopathologically advanced IgAN. Moreover, s-Gd-IgA1 levels were significantly higher in IgAN patients with glomerular sclerosis and tubulo-interstitial lesions. Mesangial-Gd-IgA1 intensity negatively correlated with eGFR in IgAN. Multivariate analysis selected s-Gd-IgA1 elevation as a significant risk factor for a 30%-reduction in eGFR in IgAN (HR, 1.37; 95% CI, 1.02–1.89; p = 0.038).

**Conclusions:**

Although IgAN and HSPN remain difficult to differentiate, s-Gd-IgA1 elevation and m-Gd-IgA1 deposition are reliable diagnostic factors that reflect IgAN severity. Serum-Gd-IgA1 could serve as a predictor of renal outcomes in IgAN. Thus, Gd-IgA1 could be significant biomarker for patients with IgAN.

## Introduction

Immunoglobulin A nephropathy (IgAN) is the most prevalent type of glomerulonephritis worldwide [1]. Progressive glomerular and interstitial sclerosis in severe IgAN leads to end stage kidney disease (ESKD) in 30%-40% of patients within 20 years after diagnosis [2, 3]. Meanwhile, 10%-20% of patients experience spontaneous remission [4, 5], which implies a variable and unpredictable clinical course of IgAN.

The gold standard for diagnosing IgAN to date has been based on evaluations of renal biopsy (RB) specimens [5], the collection of which is invasive and requires hospitalization. Moreover, evaluation of RB provides a snapshot that is not an infallible way to conclude disease severity. Therefore, findings obtained from RB specimens are presently the most incontrovertible indicator of IgAN, but a noninvasive diagnostic tool that compensates for the disadvantages of RB is desirable for patients with IgAN. Furthermore, another reliable scale for evaluating IgAN is also necessary to assure conclusions about disease activity based on RB findings.

Several clinical and histological factors have been identified as prognostic indicators for IgAN [6–10]. However, such indicators sometimes lead to inaccurate estimations of severity and long-term renal outcomes, which in turn induce misjudgments regarding the clinical management of IgAN. Undertreated patients might progress to ESKD, whereas overtreated patients might develop serious adverse events from unnecessarily intensive protocols such as steroid pulse therapy combined with tonsillectomy (TSP). Thus, a new convincing predictor would be indispensable for patients with IgAN.

Several studies have investigated aberrant IgA1 O-glycosylation, and have indicated that galactose-deficient IgA1 (Gd-IgA1) plays a pivotal role in the progression of IgAN [11–24]. Immunoglobulin A1 heavy chains generally contain a hinge region where O-glycosylation can be affected by various disorders. The O-glycosylation of IgA1 in healthy individuals requires the connection of N-acetyl-galactosamine (GalNAc) to serine or threonine residues of the hinge region, followed by the addition of galactose (Gal) to GalNac. The addition of sialic acid residues finally completes O-glycosylation [19, 25]. Patients with IgAN have aberrant IgA1 molecules with a Gal deficiency of O-linked glycans in the hinge region, which basically means Gd-IgA1 consists of terminal GalNAc or sialylated GalNAc [19, 20, 25]. These studies identified excess Gd-IgA1 in both serum and glomerular immune deposits of patients with IgAN [19, 20, 25]. Furthermore, the recently proposed multi-hit theory of IgAN states that overproduced Gd-IgA1 and autoantibodies against Gd-IgA1 subsequently form circulating immune complexes (IC), resulting in glomerular mesangial deposits followed by accelerated nephritis [19, 26]. Thus, Gd-IgA1 is vital to the pathogenesis of IgAN, and Gd-IgA1 (s-Gd-IgA1) or mesangial Gd-IgA1 (m-Gd-IgA1) could serve as candidate disease-specific biomarkers that reflect severity and prognosis. However, evidence of Gd-IgA1 as a biomarker remains controversial according to a recent meta-analysis [17]. The main reason for the controversy is the absence of a definitive assay. Serum-Gd-IgA1 can be conventionally quantified using a simple lectin-based enzyme-linked immunosorbent assay (ELISA) with a GalNAc-specific lectin from Helix aspersa (HAA), but acquiring suitable lectins has proven challenging. Moreover, HAA lectin is inappropriate for immunohistochemical analyses of m-Gd-IgA.

A novel lectin-independent ELISA assay using an anti-Gd-IgA1 monoclonal antibody (KM55) was recently developed to address the above issues [27]. Glomerular m-Gd-IgA1 deposition was confirmed by immunofluorescence (IF) with KM55 [21, 27], which provided new insights into the possibility that Gd-IgA1 could serve as a biomarker of IgAN. The present study evaluated Gd-IgA1 using KM55 and aimed to clarify the clinical significance of Gd-IgA1 in patients with IgAN.

## Patients and methods

### Study design and participants

We enrolled 111 patients who were diagnosed with IgAN based on the findings of RB specimens obtained between April 2007 and March 2017 at Showa University Hospital. Values were compared with findings from 18 patients with Henoch-Schönlein purpura nephritis (HSPN), 29 with lupus nephritis (LN), 28 with ANCA-associated vasculitis (AAV), and 13 with minimal change disease (MCD).

This historical cohort study retrospectively reviewed all enrolled patients and analyzed associations between Gd-IgA1 and patient characteristics or renal outcomes. The observation period was from April 2007 to December 2017. Renal outcome was defined as a 30% reduction in eGFR from the time of RB collection (baseline), which was a reliable surrogate endpoint [28]. All enrolled patients provided written informed consent regarding preservation of blood sample, urine sample, and kidney tissue. Also, they agreed with availability of all data obtained from RB by written informed consent. Furthermore, opt-out method was applied to obtain informed consent about measuring s-Gd-IgA1 value and evaluating m-Gd-IgA1 deposition. All enrolled patient did not refuse to participate in this study. The Ethics Committee at Showa University Hospital approved the protocol of the study (No. 2504) and the study proceeded in accordance with the ethical standards enshrined in the Declaration of Helsinki.

### Clinical and pathological parameters

Clinical characteristics, including information on follow-up period, duration from onset of abnormal urinalysis findings to the time of RB (duration from onset), age, sex, body mass index (BMI), mean arterial pressure (MAP), hematuria, urinary protein, urinary N-acetyl-beta-D-glucosaminidase (NAG) index, serum creatinine (sCr), estimated glomerular filtration rate (eGFR), serum IgA and C3, and therapeutic regimens were obtained from the records of the patients. We calculated MAP according to previous reports [29]. Hematuria was scored from 0 to 3+ as described [30]. We calculated eGFR using the modified Modification of Diet in Renal Disease (MDRD) equation for Japanese persons [31]. Therapeutic regimens, including information about treatment with renin-angiotensin system inhibitors and oral steroids, were assessed and TSP implementation was also analyzed in the patients with IgAN. Indications and the regimen for TSP are detailed elsewhere [26, 32, 33].

The clinical grade (C-grade) of IgAN at the time of RB was determined according to the criteria of the Japanese Society of Nephrology (JSN) [26, 34]. Histological sections were independently reviewed by two renal pathologists who were blinded to the clinical data of the patients. The histological severity of IgAN was determined according to the histological grading criteria of the JSN [34] or the Oxford classification [6, 7]. Risk stratification for dialysis was determined from the combination of clinical and histological grade (H-grade) according to the JSN [34] and categorized as low, medium, high, or super high risk of progression to ESKD [34] (S1 Table).

### Immunofluorescence staining

Two nephrologists independently analyzed mesangial IgA, IgG, and IgM deposits using IF according to our protocol [35] and graded the IF intensity of mesangial IgA as described [30].

### ELISA for s-Gd-IgA1

Levels of s-Gd-IgA1 were measured using sandwich ELISA kits with KM55 (#27600, Immuno-Biological Laboratories, Fujioka, Japan) [27]. Serum samples were diluted with EIA buffer (1:800), and levels were measured as recommended by the manufacturer.

### Immunohistochemistry for m-Gd-IgA1

Glomerular m-Gd-IgA1 deposition was examined by immunohistochemistry (IHC) staining as described [36, 37]. Briefly, dewaxed paraffin sections were heated with Histofine (Nichirei, Tokyo, Japan) in an autoclave at 121°C for 30 min for antigen retrieval. After endogenous peroxidase was quenched with 0.3% H_2_O_2_ in methanol, nonspecific binding was blocked with protein blocking solution and the sections were incubated overnight at 4°C with rat monoclonal anti-human Gd-IgA1 antibody (#10777, Immuno-Biological Laboratories) diluted to 1:100, followed by EnVisionTM+Dual Link System-HRP (Dako, Glostrup, Denmark) for 60 min at room temperature. Color was then developed using diaminobenzidine (DAB) (Dako).

The intensity of Gd-IgA1 in mesangial areas was assessed as 1, mild; 2, moderate; or 3, severe. Two nephrologists independently scored m-Gd-IgA1 intensity in all glomeruli in each section under × 400 magnification, and the values were averaged per section.

### Statistical analysis

Data are expressed as means ± SD or ratios (%). The results were analyzed using Prism software (GraphPad Software Inc., La Jolla, CA, USA) and JMP 9.0.1 (SAS Institute Inc., Cary, NC, USA). Non-parametric variables were compared using either Mann-Whitney U tests or Kruskal-Wallis tests. Categorical variables were compared using either Fisher exact tests or chi squared tests. Correlations between parameters were assessed using Spearman correlation coefficients, and factors favoring progression to the desired outcome were assessed by multivariate Cox regression analysis. The predictive accuracy of risk factors to discriminate outcomes was determined from receiver operating characteristic (ROC) curves and the areas under these curves (AUC).

## Results

### Characteristics of patients

Table 1 shows the baseline characteristics at the time of RB and the therapeutic regimens for 111 patients with IgAN (female, 49; mean age (± SD), 40.3 ± 14.5 years). The means (± SD) for proteinuria and eGFR were 1.3 ± 1.9 g/day and 72.2 ± 27.1 mL/min/1.73 m^2^, respectively. The proportions of M1, E1, S1, and T1-2 in the Oxford classification were 69.4%, 23.4%, 16.2%, and 16.2%, respectively. During an average follow-up of 56.6 ± 34.8 months, 35 (31.5%) patients underwent TSP.

**Table 1:** Baseline characteristics and therapeutic regimens of 111 patients with IgAN.

Characteristics	Mean ± SD or (range or percent)
Age (years)	40.3 ± 14.5 (18–75)
Male gender, No. (%)	62 (55.8)
Follow-up period (months)	56.6 ± 34.8 (10–129)
Duration from onset (months)	59.5 ± 78.1 (1–348)
BMI (kg/m^2^)	22.8 ± 4.2 (15.6–40.6)
History of hypertension^a^, No. (%)	32 (28.8)
Serum Cr level (mg/dL)	1.0 ± 0.6 (0.4–4.4)
eGFR (mL/min/1.73 mm^2^)	72.2 ± 27.1 (10.9–139.6)
Albumin (g/dL)	3.9 ± 0.6 (1.8–4.4)
Proteinuria (g/day)	1.3 ± 1.9 (0.1–10.4)
Hematuria, (±), (1+), (2+), (3+) No. (%)	8 (7.2), 13 (11.7), 16 (14.4), 74 (66.7)
Clinical grade^b^ I, II, III, No. (%)	47 (42.3), 35, (31.5), 29 (26.2)
Histological grade^b^ I, II, III, IV, No. (%)	48 (43.3), 41, (36.9), 19 (17.1), 3 (2.7)
Risk stratification for dialysis^b^	
low, medium, high, super high risk, No. (%)	33 (29.7), 37, (33.3), 32 (28.9), 9 (8.1)
Oxford classification^c^	
M 1, E 1, S1, T1-2, No. (%)	77 (69.4), 26 (23.4), 18 (16.2), 18 (16.2)
Glomerular deposition	
IgA deposition^d^: weak, moderate, strong, No. (%)	9 (8.1), 70 (63.1), 32 (28.8)
IgG deposition, No. (%)	28 (25.2)
IgM deposition, No. (%)	32 (28.8)
Electron dense deposit in capillary wall, No. (%)	9 (8.1)
Treatment after diagnosis	
Use of RASI, No. (%)	60 (54.1)
Use of antiplatelet drugs, No. (%)	75 (67.6)
Oral steroid therapy, No. (%)	54 (48.6)
Underwent Tonsillectomy, No. (%)	41 (36.9)
Underwent TSP, No. (%)	35 (31.5)

Abbreviations: No (%), number (%); BMI, body mass index; eGFR, estimated glomerular filtration rate; RASI, renin-angiotensin system inhibitor; TSP, steroid pulse therapy in combination with tonsillectomy.

^a^Blood pressure of 130/80 mmHg or higher was defined as hypertension.

^b^Clinical grade, histological grade, and risk stratification for dialysis were classified according to the criteria of the Japanese Society of Nephrology [34].

^c^Histological severity was graded according to Oxford classification [6, 7].

^d^Intensity of IgA deposition was described earlier [30].

Table 2 summarizes the outcomes of comparisons of baseline characteristics between 111 patients with IgAN and 88 patients with other types of kidney disease. The baseline characteristics did not significantly differ between IgAN and HSPN. Patients with AAV were significantly older than those with all other pathologies. The duration from onset of nephritis was significantly longer for IgAN and LN than the other groups. Although proteinuria values were comparable, patients with AAV had significantly elevated sCr and significantly decreased eGFR compared with the other groups. Serum IgA levels tended to be high in patients with IgAN, and their IgA/C3 ratios were significantly higher than those of patients with MCD.

**Table 2:** Comparison of characteristics among patients with IgAN and other kidney diseases.

Characteristics	IgAN	HSPN	LN	AAV	MCD
	(n = 111)	(n = 18)	(n = 29)	(n = 28)	(n = 13)
Age (year)	40.3 ± 14.5	46.7 ± 21.6	40.9 ± 15.6	64.7 ± 13.6^a^^,^ ^b^^,^^c^^,^ ^e^	37.6 ± 14.7
Male gender, No (%)	62 (55.8)	6 (33.3)	3 (10.3)^a^^,^ ^b^^,^ ^d^^,^ ^e^	15 (53.6)	7 (53.8)
Duration from onset (mo)	59.5 ± 78.1^b^^,^ ^d^^,^^e^	9.7 ± 12.6	81.1 ± 71.6^a^^,^ ^b^^,^ ^d^^,^ ^e^	10.3 ± 16.7	1.6 ± 0.8
BMI (kg/m^2^)	22.8 ± 4.2	21.8 ± 4.1	20.9 ± 3.1	20.4 ± 5.1	25.5 ± 5.6^c^^,^ ^d^
MAP (mmHg)	90.6 ± 12.9	87.2 ± 13.8	94.9 ± 14.1	88.8 ± 13.9	88.9 ± 21.3
Serum Cr (mg/dL)	1.0 ± 0.6	0.8 ± 0.3	0.8 ± 0.3	2.5 ± 3.0^a^^,^ ^b^^,^^c^^,^ ^e^	0.8 ± 0.2
eGFR (mL/min/1.73 mm^2^)	72.2 ± 27.1	83.2 ± 21.2	77.7 ± 27.4	38.2 ± 23.2^a^^,^ ^b^^,^^c^^,^ ^e^	87.2 ± 22.5
Alb (g/dL)	3.9 ± 0.6	3.8 ± 0.5	2.9 ± 0.9^a^	2.9 ± 0.7^a^^,^ ^b^	2.5 ± 1.6^a^^,^ ^b^
Proteinuria (g/day)	1.3 ± 2.0	0.9 ± 1.2	2.0 ± 2.3	1.2 ± 1.5	6.3 ± 7.4
Urinary NAG (U/gCr)	10.3 ± 10.4	11.2 ± 10.5	21.4 ± 18.3^a^	32.9 ± 32.9^a^^,^ ^b^	28.1 ± 34.3
Serum IgA (mg/dL)	338.0 ± 102.1	316.8 ± 87.0	291.0 ± 153.5	301.7 ± 128.8	265.4 ± 102.8
C3 (mg/dL)	105.7 ± 23.4	115.0 ± 22.2	46.9 ± 19.4^a^^,^ ^b^^,^^c^^,^ ^e^	105.6 ± 24.9	135.0 ± 24.6
CH50 (U/mL)	39.4 ± 8.6	44.8 ± 9.4	14.3 ± 12.7^a^^,^ ^b^^,^^c^^,^ ^e^	33.3 ± 12.3^b^	40.9 ± 10.2
IgA/C3	3.4 ± 1.3^e^	2.8 ± 0.6	7.1 ± 4.7^a^^,^ ^b^^,^ ^d^^,^ ^e^	2.9 ± 1.5	1.8 ± 0.6

Value are means ± SD or (percent). Mann-Whitney U test or Fisher’s test are used for statistical analysis after Kruskal-Wallis test.

^a^p<0.05 vs. IgAN group,

^b^p<0.05 vs. SHPN group,

^c^p<0.05 vs. LN group,

^d^p<0.05 vs. AAV group,

^e^p<0.05 vs. MCD group.

Abbreviations: IgAN; Immunoglobulin A nephropathy, HSPN; Henoch-Schönlein purpura nephritis, LN; lupus nephritis, AAV; ANCA-associated vasculitis, MCD; minimal change disease, No (%), number (%); mo, month; BMI, body mass index; MAP, Mean arterial pressure; Cr, creatinine; eGFR, estimated glomerular filtration rate; Alb, albumin.

### Levels of s-Gd-IgA1

**Fig 1:**
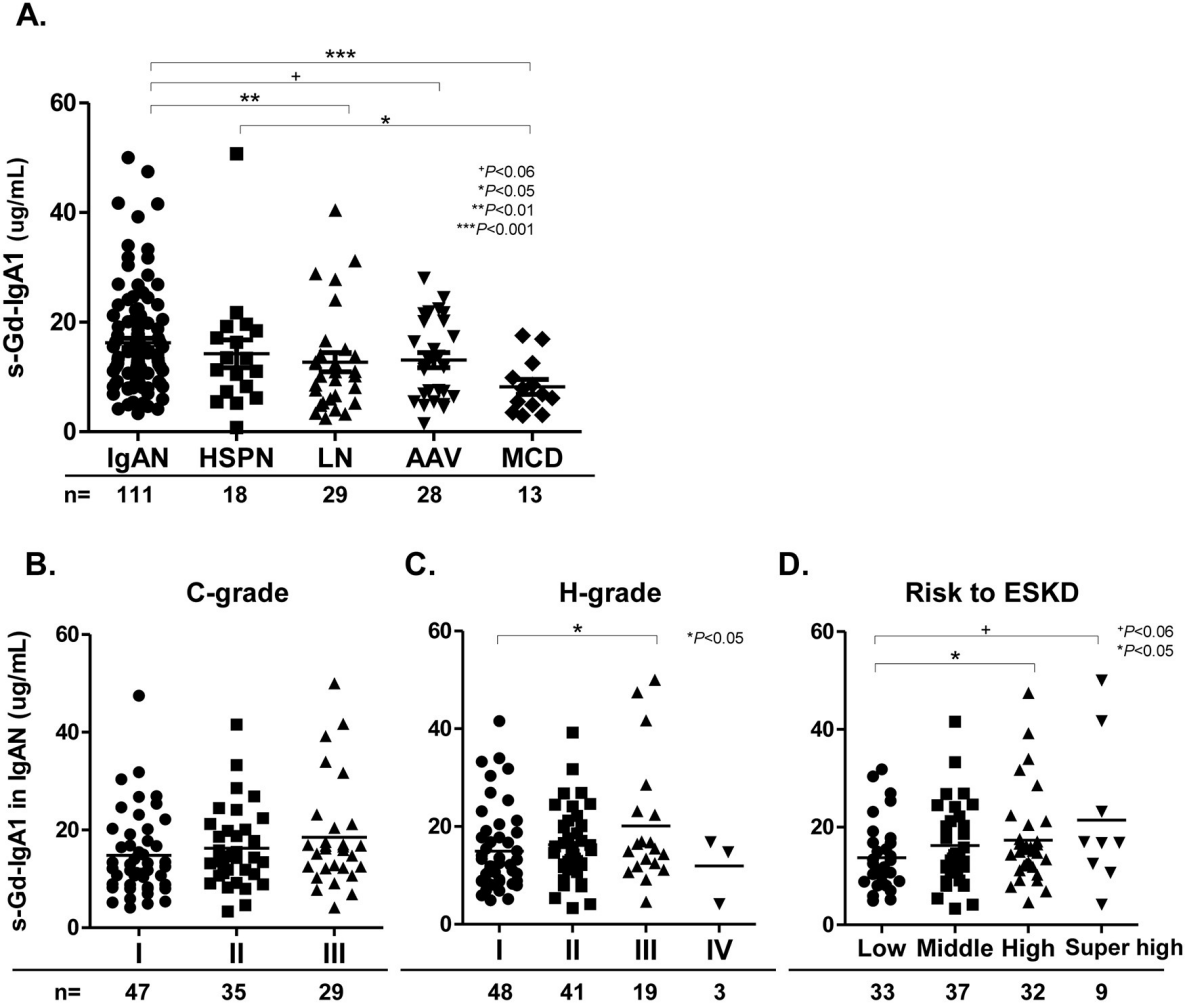
Serum-Gd-IgA1 levels determined by ELISA using KM55. Serum-Gd-IgA1 in patients with IgAN, HPN, LN, AAV, and MCD **(A)**. S-Gd-IgA1 in patients with IgAN according to JSN classification in terms of clinical grade **(B)**, histological grade **(C)**, and risk classification of progression to ESKD **(D)**. Horizontal solid lines represent means. Data were statistically analyzed using Kruskal-Wallis tests and Mann-Whitney U tests.

Fig 1A shows that s-Gd-IgA1 levels were significantly elevated in patients with IgAN compared with other kidney diseases (IgAN vs. LN, AAV, and MCD: 16.2 ± 9.1 vs. 12.7 ± 9.4, p = 0.008; 13.1 ± 7.3, p = 0.059; and 8.2 ± 4.8 μg/mL, p<0.001, respectively). Serum-Gd-IgA1 values were significantly elevated in patients with HSPN compared with MCD (14.2 ± 10.8 vs. 8.2 ± 4.8 μg/mL, p = 0.041), but did not significantly differ between IgAN and HSPN (Fig 1A).

We assigned patients with IgAN into three or four groups according to the JSN classification including C-grade, H-grade, and risk classification for ESKD. Among patients with IgAN categorized as C-grade according to value for proteinuria and eGFR, s-Gd-IgA1 values tended to be higher in those with grade III than grade I (18.5 ± 10.9 vs. 14.8 ± 8.5 μg/mL, p = 0.089) (Fig 1B). Among patients with IgAN categorized as H-grade based on the ratio of global sclerosis, segmental sclerosis, and crescents, s-Gd-IgA1 levels were significantly higher in those with grade III than grade I (20.1 ± 12.9 vs. 14.9 ± 8.5 μg/mL, p = 0.031) (Fig 1C). Consequently, s-Gd-IgA1 levels were significantly higher in patients at high or super high risk compared with those at low risk for ESKD (17.3 ± 9.5 or 21.4 ± 14.9 vs. 13.7 ± 7.1 μg/mL, p = 0.032 and p = 0.052, respectively) (Fig 1D).

### Glomerular m-Gd-IgA1 deposition

**Fig 2:**
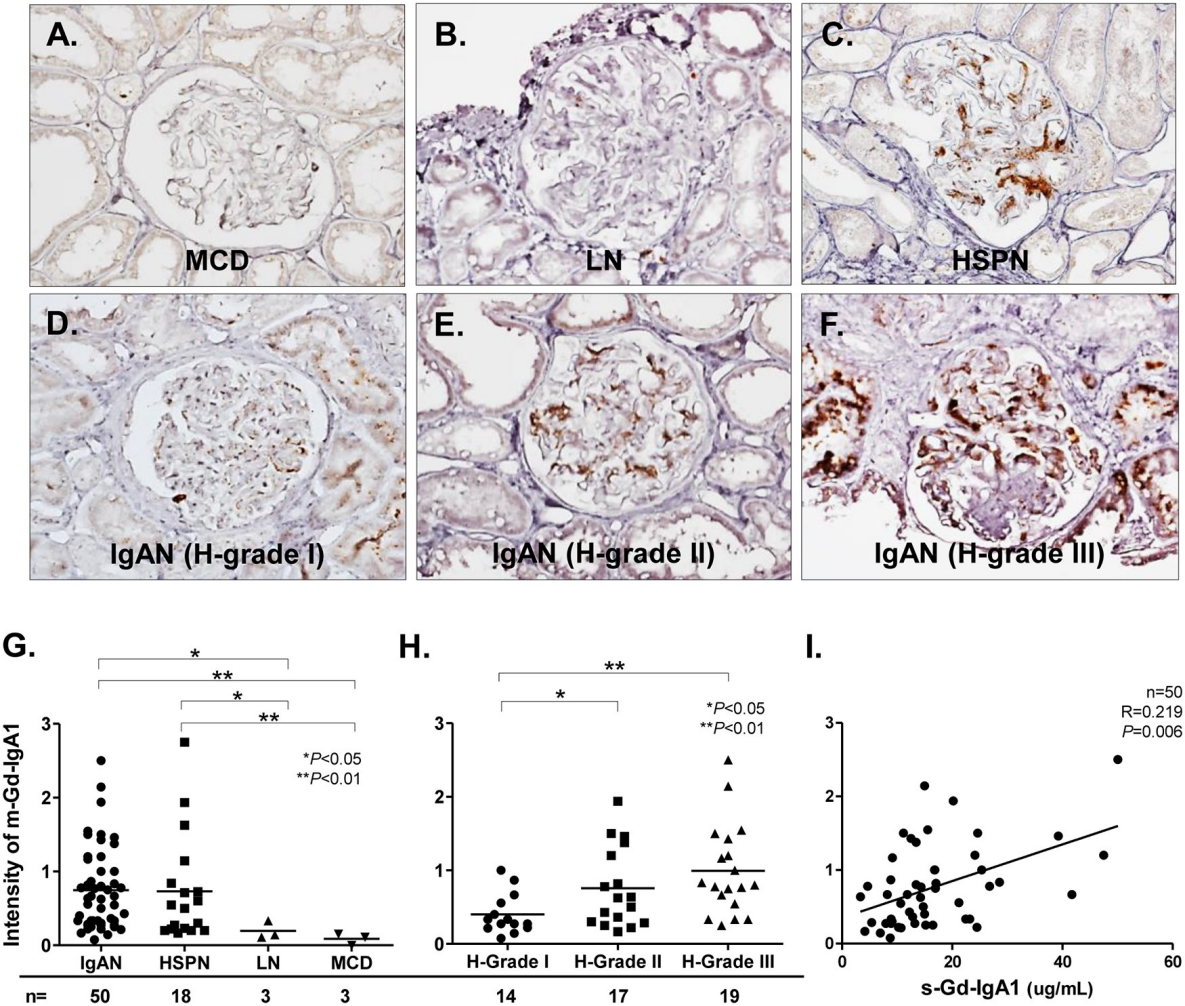
Glomerular m-Gd-IgA1 deposition identified by IHC staining for KM55. Representative photos of glomerular m-Gd-IgA1 (brown reaction product) in patients with MCD **(A)**, LN **(B)**, HSPN **(C)**, and IgAN **(D-F)**. Original magnification: ×40. Intensity of glomerular m-Gd-IgA1 compared between IgAN and other kidney diseases **(G)**. Intensity of m-Gd-IgA1 in IgAN according to JSN histological grade **(H)**. Correlation between m-Gd-IgA1 positivity and s-Gd-IgA1 level in patients with IgAN **(I)**. Horizontal solid lines represent means. Data were statistically analyzed using Kruskal-Wallis tests, Mann-Whitney U tests, and Spearman correlations.

We randomly selected 50 patients with IgAN, 18 with HSPN, 3 with LN, and 3 with MCD from the enrolled cohort to evaluate IHC staining for Gd-IgA1. Fig 2A–2F shows representative images of glomerular m-Gd-IgA1 deposition and the intensity of m-Gd-IgA1 among the selected groups. Mesangial-Gd-IgA1 deposition was apparently specific to IgAN (Fig 2D–2F) and HSPN (Fig 2C) at higher intensity, and m-Gd-IgA1 staining was more intense in these groups than in LN or MCD (IgAN vs. LN and MCD: 0.7 ± 0.6 vs. 0.2 ± 0.1, p = 0.048; 0.1 ± 0.1, p = 0.006, respectively) (HSPN vs. LN and MCD: 0.7 ± 0.7 vs. 0.2 ± 0.1, p = 0.014; 0.1 ± 0.1, p = 0.007, respectively) (Fig 2G). Staining for m-Gd-IgA1 was more intense among patients with H-grades II or III than I according to the JSN classification (0.8 ± 0.5 or 1.0 ± 0.6 vs. 0.4 ± 0.3, p = 0.029 and p<0.001, respectively) (Fig 2H). Additionally, m-Gd-IgA1 intensity scores positively correlated with s-Gd-IgA1 values in the 50 patients with IgAN (r = 0.219, p = 0.006) (Fig 2I).

### Association of Gd-IgA1 with IgAN progression

**Fig 3:**
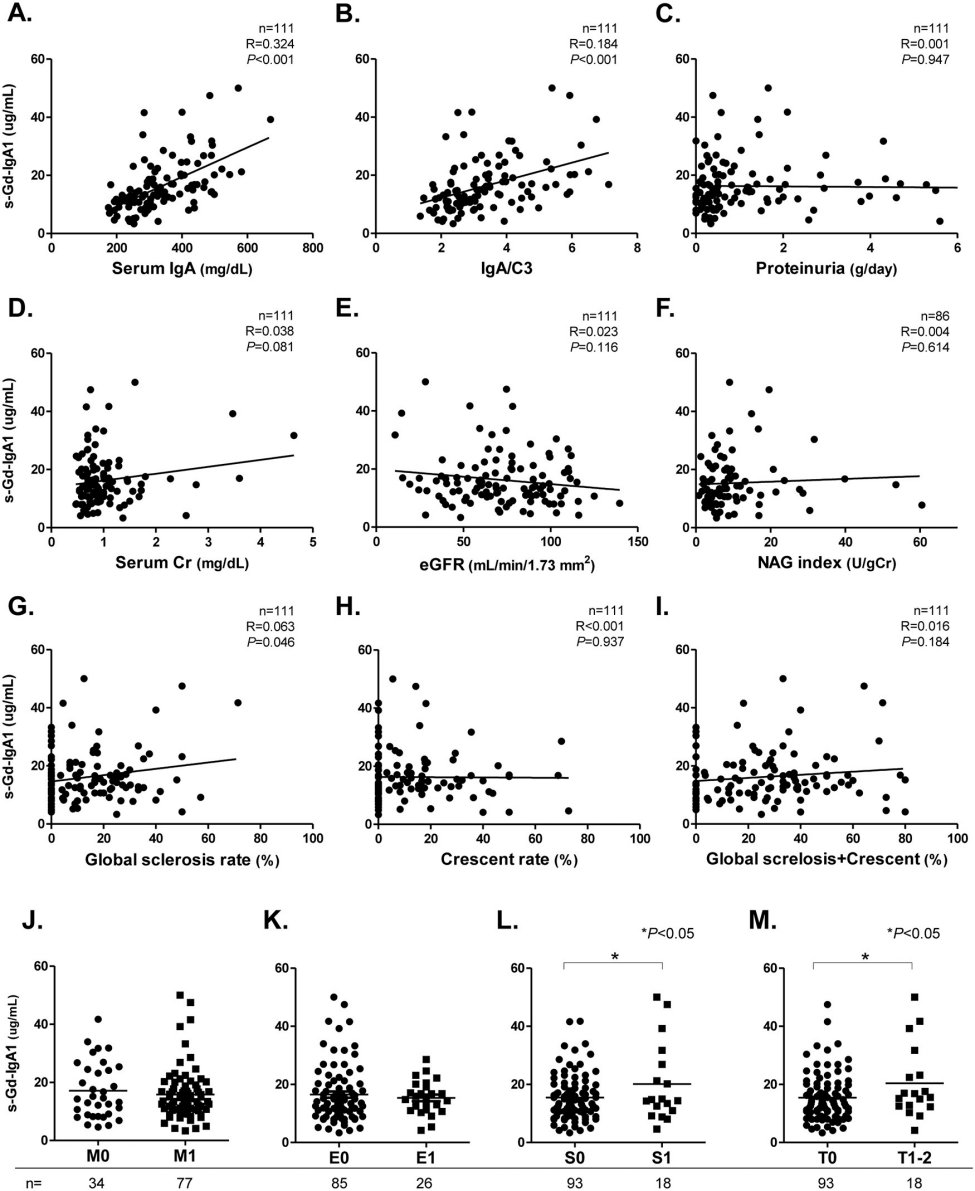
Correlations between s-Gd-IgA1 and laboratory parameters or pathological findings in patients with IgAN. Scatter plots of s-Gd-IgA1 vs. serum IgA **(A)**, serum IgA/C3 ratio **(B)**, proteinuria **(C)**, sCr **(D)**, eGFR **(E)**, urinary NAG index **(F)**, global sclerosis **(G)**, crescents **(H)**, and global sclerosis and crescents **(I)**. Rates of global sclerosis, crescents, and both types of glomerular lesions (%) were calculated by dividing total number of each type of lesion by total number of glomeruli. Crescents comprise cellular, fibrocellular, and fibrous types. S-Gd-IgA1 levels were compared based on Oxford classification (**J-M**). Patients with IgAN were assigned to groups according to mesangial hypercellularity (**J**), endocapillary hypercellularity (**K**), segmental glomerulosclerosis (**L**) and tubular atrophy/interstitial fibrosis (**M**). Horizontal solid lines represent means. Data were statistically analyzed using Mann-Whitney U tests and Spearman correlation tests.

**Fig 4:**
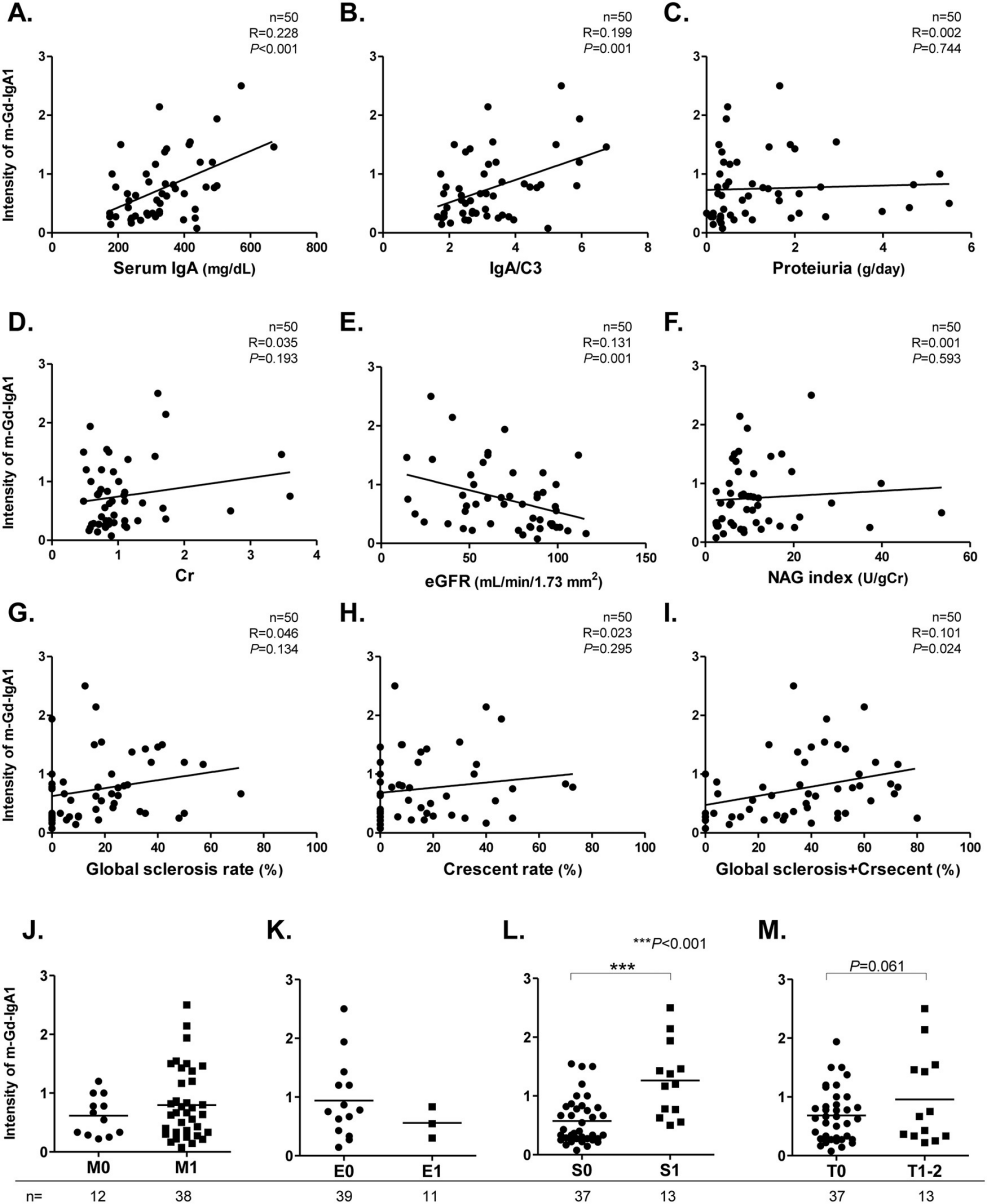
Correlations between m-Gd-IgA1 deposition and laboratory parameters or pathological findings in patients with IgAN. Scatter plots of m-Gd-IgA1 intensity vs. serum IgA (**A**), serum IgA/C3 ratio (**B**), proteinuria (**C**), sCr (**D**), eGFR (**E**), urinary NAG index (**F**), global sclerosis (**G**), crescents (**H**), and global sclerosis and crescents (**I**). Rates of global sclerosis, crescents, and both glomerular lesions (%) were calculated by dividing total numbers of each lesion by otal number of glomeruli. Crescents comprised cellular, fibrocellular, and fibrous types. Mesangial-Gd-IgA1 intensity was compared based on Oxford classification (**J-M**). Patients with IgAN were assigned to groups according to mesangial hypercellularity (**J**), endocapillary hypercellularity (**K**), segmental glomerulosclerosis (**L**), and tubular atrophy/interstitial fibrosis (**M**). Horizontal solid lines represent means. Data were statistically analyzed using Mann-Whitney U tests and Spearman correlation tests.

Fig 3 shows associations between s-Gd-IgA1 levels and laboratory parameters or pathological findings in 111 patients with IgAN. Fig 4 shows associations between m-Gd-IgA1 intensity and clinical parameters in 50 patients. In terms of basic data, serum IgA and IgA/C3 ratios positively correlated with s-Gd-IgA1 (r = 0.324, p<0.001) (r = 0.184, p<0.001) (Fig 3A and 3B) or m-Gd-IgA1 (r = 0.228, p<0.001) (r = 0.199, p<0.001) (Fig 4A and 4B). However, neither s-Gd-IgA1 nor m-d-IgA1 significantly correlated with any other basic parameters such as age, duration from onset, and MAP (S1 Fig).

Considering renal function, s-Gd-IgA1 tended to correlate with sCr values (r = 0.038, p = 0.081) (Fig 3D) and global sclerosis rates (r = 0.063, p = 0.046) (Fig 3G). Furthermore, m-Gd-IgA1 correlated significantly and negatively with eGFR (r = 0.131, p = 0.001) (Fig 4E) and positively with global sclerosis plus crescent rates (r = 0.101, p = 0.024) (Fig 4I). However, neither type of Gd-IgA1 correlated with proteinuria or crescent rates. Based on the Oxford classification, s-Gd-IgA1 values were significantly higher in patients with segmental sclerosis and tubular atrophy/interstitial fibrosis (S0 vs. S1: 15.5 ± 7.8 vs. 20.1 ± 13.6 μg/mL, p = 0.023) (T0 vs. T1-2: 15.4 ± 8.1 vs. 20.4 ± 12.4 μg/mL, p = 0.017) (Fig 3L and 3M), and m-Gd-IgA1 intensity was similar (S0 vs. S1: 0.6 ± 0.4 vs. 1.3 ± 0.6, p<0.001) (T0 vs. T1-2: 0.7 ± 0.4 vs. 0.9 ± 0.8, p = 0.061) (Fig 4L and 4M).

### Multivariate analysis of factors contributing to renal outcome in IgAN

During the observation period of 111 patients with IgAN, eGFR declined by 30% from baseline in 16 patients (14.4%). Table 3 summarizes the hazard ratios (HR) of possible factors related to outcomes and the 95% confidence intervals (CI) for these patients. The findings of univariate analysis showed that s-Gd-IgA1 elevation increased the HR for a 30% reduction in eGFR (HR, 1.34; 95% CI, 1.09–1.62; p = 0.008) (Table 3) as well as an increase in sCr, an elevated IgA/C3 ratio, and more advanced H-grade. Multivariate analysis selected elevated sCr or IgA/C3 ratios as independent risk factors for a decline in eGFR. The results of multivariate analysis similarly showed that s-Gd-IgA1 elevation worsened the HR for a 30% reduction in eGFR (HR 1.37; 95% CI, 1.02–1.89; p = 0.038) (Table 3). Furthermore, we calculated the AUC (S2 Fig) to evaluate the predictive accuracy of s-Gd-IgA1 for discriminating a 30% reduction in eGFR. According to the ROC curves and AUC = 0.77 (p = 0.002), the threshold of s-Gd-IgA1 for predicting a 30% reduction in eGFR was 23.2 μg/mL with 56.0% sensitivity and 88.4% specificity.

**Table 3:** Univariate and multivariate analysis of possible factors that contributed to 30% eGFR reduction in 111 patients with IgAN.

Variable	Univariate analysis			Multivariate analysis		
	HR	95% CI	P Value	HR	95% CI	P Value
Age (per 10 year of age)	0.94	(0.62–1.38)	0.777	0.64	(0.36–1.07)	0.092
Male gender (vs. female)	1.83	(0.67–5.39)	0.238	0.59	(0.10–3.37)	0.550
MAP (per 10 mmHg)	1.31	(0.91–1.86)	0.151	1.09	(0.58–1.95)	0.767
Serum Cr (per 0.5 mg/dL)	1.44	(1.09–1.78)	0.011*	1.99	(1.03–3.85)	0.039*
Proteinuria (per 0.5 g/day)	1.12	(0.99–1.24)	0.062	1.18	(0.98–1.38)	0.079
eGFR (per 10 mL/min/1.73m^2^)	0.83	(0.67–1.02)	0.078	1.28	(0.82–1.97)	0.273
IgA/C3 ratio (per 1)	1.68	(1.16–2.39)	0.007**	1.78	(1.09–2.89)	0.022*
Serum Gd-IgA1 (per 5 μg/mL)	1.34	(1.09–1.62)	0.008**	1.37	(1.02–1.89)	0.038*
Histological grade^a^ (per grade)	2.38	(1.35–4.22)	0.028*	0.99	(0.38–2.63)	0.795
Use of RASI (vs. absence)	1.69	(0.61–5.38)	0.320	2.23	(0.35–14.46)	0.395
Underwent TSP (vs. absence)	1.72	(0.53–4.97)	0.345	0.84	(0.17–3.54)	0.828

Abbreviations: HR, hazard ratio; CI, confidence interval; MAP, mean arterial pressure; Cr, creatinine; eGFR, estimated glomerular filtration rate; RASI, renin-angiotensin system inhibitor; TSP, steroid pulse therapy in combination with tonsillectomy.

^a^Histological grade was classified according to the criteria of the Japanese Society of Nephrology [34].

*P<0.05,

**P<0.01

## Discussion

We showed that KM-55 consistently detected s-Gd-IgA1 or m-Gd-IgA1 without any type of burden, which others have also indicated [21, 27]. Moreover, the present and previous data generated using ELISA kits or IF staining with KM55 did not significantly differ [21, 27]. These findings indicated that novel lectin independent methods using KM55 can reliably and reproducibly evaluate Gd-IgA1 compared with conventional HAA-lectin assays, and thus could serve as a powerful tool with which to clarify this unpredictable disease.

As far as we can ascertain, detailed information about s-Gd-IgA1 levels and m-Gd-IgA1 deposition, in IgAN, HSPN, and other kidney diseases has not been published. The present findings further clarified the relationships among Gd-IgA1, IgAN disease severity, and renal prognosis, which had hitherto been ambiguous.

With respect to diagnostic value, s-Gd-IgA1 was significantly elevated and m-Gd-IgA1 deposition was specific to IgAN, whereas s-Gd-IgA1 did not significantly differ among LN, AAV, and MCD. Staining for m-Gd-IgA1 was very faint in patients with either MCD or LN. These findings were compatible with previous results [21, 22, 27, 38, 39] and indicated that Gd-IgA1 can differentiate IgAN from other kidney diseases. However, careful interpretation of s-Gd-IgA1 values is necessary since immunosuppressive therapy with agents such as steroids could influence the results. Indeed, more patients with AAV or LN than IgAN were under immunosuppressive regimes with steroid pulse therapy, oral steroid therapy and other immunosuppressive agents (S2 Table). Furthermore, a distinction from HSPN remains obscure. The present results are similar to those of recent studies [15, 17, 40, 41], and Suzuki et al. noted that IgAN and HSPN share the feature of Gd-IgA1-oriented pathogenesis [21]. To distinguish between IgAN and HSPN will be a monumental task, but this must be elucidated. Moreover, why m-Gd-IgA1 is not deposited while s-Gd-IgA1 or its IC is detectable in patients with non-IgAN remains obscure. We detected s-Gd-IgA1 in patients with LN, AAV, and MCD. Others have identified not only s-Gd-IgA1, but also s-Gd-IgA1-IgA/IgG-IC in patients with non-IgAN and in healthy persons [5, 22]. Based on the multi-hit theory of IgAN, s-Gd-IgA1 and its IC are critical for m-Gd-IgA1-IgG/IgA IC deposition, but considering the key enzyme ST6GalNAc-II that prevents galactosylation by C1GalT1 under physiological conditions [24, 42], the formation of Gd-IgA1 and its IC is plausible, even in healthy persons. Collectively, altered sustained enzyme activities due to genetic or environmental factors could contribute to Gd-IgA1 overproduction in IgAN, thus leading to s-Gd-IgA1 elevation and m-Gd-IgA1 deposition in IgAN. Further analysis is required to determine the normal range of s-Gd-IgA1 and to distinguish patients with mild IgAN from healthy persons.

The novel finding of the present study is that both types of Gd-IgA1 reflected disease activity. Indeed, the positive correlation was significant for both types of Gd-IgA1, and s-Gd-IgA1 elevation and m-Gd-IgA1 positivity were remarkable in patients with the advanced H-grade of IgAN. Furthermore, s-Gd-IgA1 or m-Gd-IgA1 tended to correlate with renal dysfunction. Notably, a correlation with renal dysfunction was limited only to IgAN, because s-Gd-IgA1 in LN or AAV did not correlate with renal dysfunction (S3 Fig). Levels of s-Gd-IgA1 even after correction by sCr levels were also significantly higher in IgAN or HSPN than in other kidney diseases (S4 Fig). These findings could dispel the notion that s-Gd-IgA1 elevation in IgAN is attributable simply to a decline in glomerular clearance due to renal dysfunction. However, the findings of a recent systematic review were contrary to ours [17], and s-Gd-IgA1 was not associated with clinical manifestations and pathological grade in children with IgAN [43]. Meanwhile, Nakata et al. reported a significant reduction in s-Gd-IgA1 with remarkable improvement of urinary abnormalities after TSP [44]. Berthelot et al. suggested that both s-Gd-IgA1 and m-Gd-IgA1 can predict IgAN recurrence in kidney grafts [45]. These findings indicated that renal injury in IgAN is linked to fluctuating s-Gd-IgA1 levels and m-Gd-IgA1 intensity, which supports the potential of Gd-IgA1 as an activity marker of IgAN. However, few results generated using KM55 have been published, and further prospective studies using this novel assay will be indispensable.

Another novel finding of the present study is that Gd-IgA1 did not correlate with either proteinuria or crescent formation, which are common during the acute phase of IgAN. Our results for proteinuria were similar to those of Hastings et al. [46]. However, others have significantly associated the degree of proteinuria with not only s-Gd-IgA1 [5, 47] but also urinary Gd-IgA1 [18] or s-Gd-IgA1-IgG/IgA IC [22]. One recent study found that a higher degree of galactose deficiency was associated with urinary IgA1 than serum IgA1 [18], indicating that urinary Gd-IgA1 might be more sensitive for evaluating acute IgAN lesions. Furthermore, many reports have emphasized the clinical value of s-Gd-IgA1-specific antibodies rather than s-Gd-IgA1 itself. Levels of s-Gd-IgA1-IgG/IgA IC were significantly higher in patients with large numbers of crescents or large amounts of mesangial IgA or IgG deposition [22]. We did not assess s-Gd-IgA1-specific antibodies due to the consideration that this procedure is inappropriate for clinical applications because sample preparation is complicated. Additionally, neither type of Gd-IgA1 was associated with mesangial IgA intensity or mesangial IgG deposition (S5 Fig). Thus, urinary Gd-IgA1 or specific antibodies to s-Gd-IgA1 might be suitable markers of acute lesions in IgAN. Intensive analysis focusing on all parameters related to Gd-IgA1-formation in the multi-hit theory is desirable to summarize the diverse results of various investigations including the present study.

Similarly to s-Gd-IgA1 formation, incidental mesangial IgA deposition can be encountered in healthy individuals [48], which conversely implies that longitudinal and sustainable stimulation originating from excessive s-Gd-IgA is essential for progressive nephritis. That is, Gd-IgA1 might affect the progression of IgAN over a long period. Consequently, we confirmed significant associations between Gd-IgA1 and chronic lesions such as glomerular sclerosis or tubulo-interstitial lesions, and patients with IgAN who have high s-Gd-IgA1 levels tended to have a poor renal prognosis. Few reports have described the potential of Gd-IgA1 as a prognostic factor [23, 47, 49]. The present findings and the results of these studies suggest that Gd-IgA1 could help to predict patients with IgAN who will have a poor prognosis and require intensive treatment.

Our study had several limitations. First, follow-up was short. Second, we collected data only at the time of RB. Third, we did not investigate true endpoints based on renal survival rates. More data are needed to generate additional concrete evidence of the value of Gd-IgA1 as a real-time biomarker.

In conclusion, s-Gd-IgA1 elevation and m-Gd-IgA1 deposition comprise a reliable diagnostic indicator for IgAN, although the present study could not clarify a distinction from HSPN. Gd-IgA1 notably correlates with chronic lesions and reflects disease severity, and s-Gd-IgA1 has the potential to predict renal outcomes. Thus, evaluating Gd-IgA1 is important, as it could serve as a valuable biomarker for patients with IgAN.

## Supporting information

S1 Fig Correlations between s-Gd-IgA1 level or m-Gd-IgA1 intensity and basic parameters in patients with IgAN. Scatter plots of s-Gd-IgA1 values or m-Gd-IgA1 intensity vs. age (A and D), duration from onset (B and E), and MAP (C and F). Data were statistically analyzed using Spearman correlation tests.
(PDF)

S2 Fig Renal outcomes of patients with IgAN determined from ROC curves of s-Gd-IgA1. Receiver operator characteristic curves and calculation of AUC for s-Gd-IgA1 value required to predict 30% eGFR reduction in patients with IgAN (n = 111).
(PDF)

S3 Fig Correlation between s-Gd-IgA1 levels and renal function in patients with kidney diseases other than IgAN. Scatter plots show s-Gd-IgA1 vs. sCr and eGFR in patients with HSPN (A and E), LN (B and F), AAV (C and G), and MCD (D and H). Data were statistically analyzed using Spearman correlation tests.
(PDF)

S4 Fig Levels of s-Gd-IgA1 after correction for sCr levels. Serum-Gd-IgA1 values divided by sCr values for individual patient were compared among the study groups. Data were statistically analyzed using Mann-Whitney U tests.
(PDF)

S5 Fig Comparison of s-Gd-IgA1 or m-Gd-IgA1 according to mesangial immune-complex depositions in patients with IgAN. Patients were assigned to groups according to mesangial IgA intensity (A and D), IgG deposition (B and E), and IgM deposition (C and F), then compared with s-Gd-IgA1 values or m-Gd-IgA1 intensity. Horizontal solid lines represent means. Data were statistically analyzed using Kruskal-Wallis tests and Mann-Whitney U tests.
(PDF)

S1 Table Risk stratification for dialysis according to the criteria of the Japanese Society of Nephrology. (RTF)

S2 Table Comparison of immunosuppressive treatment among study groups when renal biopsies were obtained. (RTF)

## References

[1] McGrogan A, Franssen CF, de Vries CS. The incidence of primary glomerulonephritis worldwide: a systematic review of the literature. Nephrol Dial Transplant. 2011; 26(2):414–30. 10.1093/ndt/gfq665 . 21068142

[2] Coppo R. Clinical and histological risk factors for progression of IgA nephropathy: an update in children, young and adult patients. J Nephrol. 2017; 30(3):339–46. 10.1007/s40620-016-0360-z . 27815919

[3] Gutierrez E, Zamora I, Ballarin JA, Arce Y, Jimenez S, Quereda C, et al Long-term outcomes of IgA nephropathy presenting with minimal or no proteinuria. J Am Soc Nephrol. 2012; 23(10):1753–60. 10.1681/ASN.2012010063 . 22956820 PMC3458461

[4] Imai H, Miura N. A treatment dilemma in adult immunoglobulin A nephropathy: what is the appropriate target, preservation of kidney function or induction of clinical remission? Clin Exp Nephrol. 2012; 16(2):195–201. 10.1007/s10157-011-0552-8 . 22086123 PMC3328677

[5] Suzuki Y, Matsuzaki K, Suzuki H, Okazaki K, Yanagawa H, Ieiri N, et al Serum levels of galactose-deficient immunoglobulin (Ig) A1 and related immune complex are associated with disease activity of IgA nephropathy. Clin Exp Nephrol. 2014; 18(5):770–7. 10.1007/s10157-013-0921-6 . 24477513 PMC4194014

[6] Cattran DC, Coppo R, Cook HT, Feehally J, Roberts IS, Troyanov S, et al The Oxford classification of IgA nephropathy: rationale, clinicopathological correlations, and classification. Kidney Int. 2009; 76(5):534–45. 10.1038/ki.2009.243 . 19571791

[7] Roberts IS, Cook HT, Troyanov S, Alpers CE, Amore A, Barratt J, et al The Oxford classification of IgA nephropathy: pathology definitions, correlations, and reproducibility. Kidney Int. 2009; 76(5):546–56. 10.1038/ki.2009.168 . 19571790

[8] Coppo R, Lofaro D, Camilla RR, Bellur S, Cattran D, Cook HT, et al Risk factors for progression in children and young adults with IgA nephropathy: an analysis of 261 cases from the VALIGA European cohort. Pediatr Nephrol. 2017; 32(1):139–50. 10.1007/s00467-016-3469-3 . 27557557

[9] Coppo R, D'Amico G. Factors predicting progression of IgA nephropathies. J Nephrol. 2005; 18(5):503–12. . 16299675

[10] Kawamura T, Joh K, Okonogi H, Koike K, Utsunomiya Y, Miyazaki Y, et al A histologic classification of IgA nephropathy for predicting long-term prognosis: emphasis on end-stage renal disease. J Nephrol. 2013; 26(2):350–7. 10.5301/jn.5000151 . 22684645

[11] Hastings MC, Moldoveanu Z, Julian BA, Novak J, Sanders JT, McGlothan KR, et al Galactose-deficient IgA1 in African Americans with IgA nephropathy: serum levels and heritability. Clin J Am Soc Nephrol. 2010; 5(11):2069–74. 10.2215/CJN.03270410 . 20634323 PMC3001782

[12] Kim MJ, Schaub S, Molyneux K, Koller MT, Stampf S, Barratt J. Effect of Immunosuppressive Drugs on the Changes of Serum Galactose-Deficient IgA1 in Patients with IgA Nephropathy. PLoS One. 2016; 11(12):e0166830 10.1371/journal.pone.0166830 adherence to PLOS ONE policies on sharing data and materials. 27930655 PMC5145158

[13] Kiryluk K, Li Y, Moldoveanu Z, Suzuki H, Reily C, Hou P, et al GWAS for serum galactose-deficient IgA1 implicates critical genes of the O-glycosylation pathway. PLoS Genet. 2017; 13(2):e1006609 10.1371/journal.pgen.1006609 . 28187132 PMC5328405

[14] Moldoveanu Z, Wyatt RJ, Lee JY, Tomana M, Julian BA, Mestecky J, et al Patients with IgA nephropathy have increased serum galactose-deficient IgA1 levels. Kidney Int. 2007; 71(11):1148–54. 10.1038/sj.ki.5002185 . 17342176

[15] Pillebout E, Jamin A, Ayari H, Housset P, Pierre M, Sauvaget V, et al Biomarkers of IgA vasculitis nephritis in children. PLoS One. 2017; 12(11):e0188718 10.1371/journal.pone.0188718 . 29190714 PMC5708800

[16] Placzek WJ, Yanagawa H, Makita Y, Renfrow MB, Julian BA, Rizk DV, et al Serum galactose-deficient-IgA1 and IgG autoantibodies correlate in patients with IgA nephropathy. PLoS One. 2018; 13(1):e0190967 10.1371/journal.pone.0190967 . 29324897 PMC5764330

[17] Sun Q, Zhang Z, Zhang H, Liu X. Aberrant IgA1 Glycosylation in IgA Nephropathy: A Systematic Review. PLoS One. 2016; 11(11):e0166700 10.1371/journal.pone.0166700 . 27870872 PMC5117702

[18] Suzuki H, Allegri L, Suzuki Y, Hall S, Moldoveanu Z, Wyatt RJ, et al Galactose-Deficient IgA1 as a Candidate Urinary Polypeptide Marker of IgA Nephropathy? Dis Markers. 2016; 2016:7806438 10.1155/2016/7806438 . 27647947 PMC5018335

[19] Suzuki H, Kiryluk K, Novak J, Moldoveanu Z, Herr AB, Renfrow MB, et al The pathophysiology of IgA nephropathy. J Am Soc Nephrol. 2011; 22(10):1795–803. 10.1681/ASN.2011050464 . 21949093 PMC3892742

[20] Suzuki H, Moldoveanu Z, Hall S, Brown R, Vu HL, Novak L, et al IgA1-secreting cell lines from patients with IgA nephropathy produce aberrantly glycosylated IgA1. J Clin Invest. 2008; 118(2):629–39. 10.1172/JCI33189 . 18172551 PMC2157566

[21] Suzuki H, Yasutake J, Makita Y, Tanbo Y, Yamasaki K, Sofue T, et al IgA nephropathy and IgA vasculitis with nephritis have a shared feature involving galactose-deficient IgA1-oriented pathogenesis. Kidney Int. 2018; 93(3):700–5. 10.1016/j.kint.2017.10.019 . 29329643

[22] Yanagawa H, Suzuki H, Suzuki Y, Kiryluk K, Gharavi AG, Matsuoka K, et al A panel of serum biomarkers differentiates IgA nephropathy from other renal diseases. PLoS One. 2014; 9(5):e98081 10.1371/journal.pone.0098081 . 24858067 PMC4032235

[23] Zhao N, Hou P, Lv J, Moldoveanu Z, Li Y, Kiryluk K, et al The level of galactose-deficient IgA1 in the sera of patients with IgA nephropathy is associated with disease progression. Kidney Int. 2012; 82(7):790–6. 10.1038/ki.2012.197 . 22673888 PMC3443545

[24] Suzuki H, Fan R, Zhang Z, Brown R, Hall S, Julian BA, et al Aberrantly glycosylated IgA1 in IgA nephropathy patients is recognized by IgG antibodies with restricted heterogeneity. J Clin Invest. 2009; 119(6):1668–77. 10.1172/JCI38468 . 19478457 PMC2689118

[25] Kiryluk K, Novak J. The genetics and immunobiology of IgA nephropathy. J Clin Invest. 2014; 124(6):2325–32. 10.1172/JCI74475 . 24892706 PMC4089454

[26] Tomino Y. Diagnosis and treatment of patients with IgA nephropathy in Japan. Kidney Res Clin Pract. 2016; 35(4):197–203. 10.1016/j.krcp.2016.09.001 . 27957413 PMC5142264

[27] Yasutake J, Suzuki Y, Suzuki H, Hiura N, Yanagawa H, Makita Y, et al Novel lectin-independent approach to detect galactose-deficient IgA1 in IgA nephropathy. Nephrol Dial Transplant. 2015; 30(8):1315–21. 10.1093/ndt/gfv221 . 26109484 PMC4513896

[28] Coresh J, Turin TC, Matsushita K, Sang Y, Ballew SH, Appel LJ, et al Decline in estimated glomerular filtration rate and subsequent risk of end-stage renal disease and mortality. JAMA. 2014; 311(24):2518–31. 10.1001/jama.2014.6634 . 24892770 PMC4172342

[29] Razminia M, Trivedi A, Molnar J, Elbzour M, Guerrero M, Salem Y, et al Validation of a new formula for mean arterial pressure calculation: the new formula is superior to the standard formula. Catheter Cardiovasc Interv. 2004; 63(4):419–25. 10.1002/ccd.20217 . 15558774

[30] Wada Y, Ogata H, Takeshige Y, Takeshima A, Yoshida N, Yamamoto M, et al Clinical significance of IgG deposition in the glomerular mesangial area in patients with IgA nephropathy. Clin Exp Nephrol. 2013; 17(1):73–82. 10.1007/s10157-012-0660-0 . 22752397 PMC3572378

[31] Imai E, Horio M, Nitta K, Yamagata K, Iseki K, Tsukamoto Y, et al Modification of the Modification of Diet in Renal Disease (MDRD) Study equation for Japan. Am J Kidney Dis. 2007; 50(6):927–37. 10.1053/j.ajkd.2007.09.004 . 18037093

[32] Murakoshi M, Gohda T, Sonoda Y, Suzuki H, Tomino Y, Horikoshi S, et al Effect of tonsillectomy with steroid pulse therapy on circulating tumor necrosis factor receptors 1 and 2 in IgA nephropathy. Clin Exp Nephrol. 2017; 21(6):1068–74. 10.1007/s10157-017-1408-7 . 28389814

[33] Kawamura T, Yoshimura M, Miyazaki Y, Okamoto H, Kimura K, Hirano K, et al A multicenter randomized controlled trial of tonsillectomy combined with steroid pulse therapy in patients with immunoglobulin A nephropathy. Nephrol Dial Transplant. 2014; 29(8):1546–53. 10.1093/ndt/gfu020 . 24596084 PMC4106640

[34] Matsuo S. Clinical guides for immunoglobulin A (IgA) nephropathy in Japan, third version. Jpn J Nephrol. 2011; 53(2):123–35. . 21516693

[35] Hihara K, Iyoda M, Tachibana S, Iseri K, Saito T, Yamamoto Y, et al Anti-Phospholipase A2 Receptor (PLA2R) Antibody and Glomerular PLA2R Expression in Japanese Patients with Membranous Nephropathy. PLoS One. 2016; 11(6):e0158154 10.1371/journal.pone.0158154 . 27355365 PMC4927164

[36] Iyoda M, Shibata T, Hirai Y, Kuno Y, Akizawa T. Nilotinib attenuates renal injury and prolongs survival in chronic kidney disease. J Am Soc Nephrol. 2011; 22(8):1486–96. 10.1681/ASN.2010111158 . 21617123 PMC3148703

[37] Wada Y, Iyoda M, Matsumoto K, Shindo-Hirai Y, Kuno Y, Yamamoto Y, et al Epidermal growth factor receptor inhibition with erlotinib partially prevents cisplatin-induced nephrotoxicity in rats. PLoS One. 2014; 9(11):e111728 10.1371/journal.pone.0111728 . 25390346 PMC4229108

[38] Shimozato S, Hiki Y, Odani H, Takahashi K, Yamamoto K, Sugiyama S. Serum under-galactosylated IgA1 is increased in Japanese patients with IgA nephropathy. Nephrol Dial Transplant. 2008; 23(6):1931–9. 10.1093/ndt/gfm913 . 18178603

[39] Sanders JT, Hastings MC, Moldoveanu Z, Novak J, Julian BA, Bursac Z, et al Serial Galactose-Deficient IgA1 Levels in Children with IgA Nephropathy and Healthy Controls. Int J Nephrol. 2017; 2017:8210641 10.1155/2017/8210641 . 29333295 PMC5733148

[40] Heineke MH, Ballering AV, Jamin A, Ben Mkaddem S, Monteiro RC, Van Egmond M. New insights in the pathogenesis of immunoglobulin A vasculitis (Henoch-Schonlein purpura). Autoimmun Rev. 2017; 16(12):1246–53. 10.1016/j.autrev.2017.10.009 . 29037908

[41] Berthelot L, Jamin A, Viglietti D, Chemouny JM, Ayari H, Pierre M, et al Value of biomarkers for predicting immunoglobulin A vasculitis nephritis outcome in an adult prospective cohort. Nephrol Dial Transplant. 2017 10.1093/ndt/gfx300 . 29126311

[42] Suzuki H, Raska M, Yamada K, Moldoveanu Z, Julian BA, Wyatt RJ, et al Cytokines alter IgA1 O-glycosylation by dysregulating C1GalT1 and ST6GalNAc-II enzymes. J Biol Chem. 2014; 289(8):5330–9. 10.1074/jbc.M113.512277 . 24398680 PMC3931088

[43] Jiang M, Jiang X, Rong L, Xu Y, Chen L, Qiu Z, et al Serum galactose-deficient IgA1 levels in children with IgA nephropathy. Int J Clin Exp Med. 2015; 8(5):7861–6. . 26221341 PMC4509286

[44] Nakata J, Suzuki Y, Suzuki H, Sato D, Kano T, Yanagawa H, et al Changes in nephritogenic serum galactose-deficient IgA1 in IgA nephropathy following tonsillectomy and steroid therapy. PLoS One. 2014; 9(2):e89707 10.1371/journal.pone.0089707 . 24586974 PMC3931817

[45] Berthelot L, Robert T, Vuiblet V, Tabary T, Braconnier A, Drame M, et al Recurrent IgA nephropathy is predicted by altered glycosylated IgA, autoantibodies and soluble CD89 complexes. Kidney Int. 2015; 88(4):815–22. 10.1038/ki.2015.158 . 26061544

[46] Hastings MC, Afshan S, Sanders JT, Kane O, Eison TM, Lau KK, et al Serum galactose-deficient IgA1 level is not associated with proteinuria in children with IgA nephropathy. Int J Nephrol. 2012; 2012:315467. 10.1155/2012/315467 . 22754697 PMC3382943

[47] Camilla R, Suzuki H, Dapra V, Loiacono E, Peruzzi L, Amore A, et al Oxidative stress and galactose-deficient IgA1 as markers of progression in IgA nephropathy. Clin J Am Soc Nephrol. 2011; 6(8):1903–11. 10.2215/CJN.11571210 . 21784819 PMC3156425

[48] Suzuki K, Honda K, Tanabe K, Toma H, Nihei H, Yamaguchi Y. Incidence of latent mesangial IgA deposition in renal allograft donors in Japan. Kidney Int. 2003; 63(6):2286–94. 10.1046/j.1523-1755.63.6s.2.x . 12753320

[49] Berthoux F, Suzuki H, Thibaudin L, Yanagawa H, Maillard N, Mariat C, et al Autoantibodies targeting galactose-deficient IgA1 associate with progression of IgA nephropathy. J Am Soc Nephrol. 2012; 23(9):1579–87. 10.1681/ASN.2012010053 . 22904352 PMC3431415

