# Supplementary figures and images for: Clinical significance of serum and mesangial galactose-deficient IgA1 in patients with IgA nephropathy
Source: PLoS One. 2018 Nov 2;13(11):e0206865. doi: 10.1371/journal.pone.0206865 (PMC6214568; doi:10.1371/journal.pone.0206865)

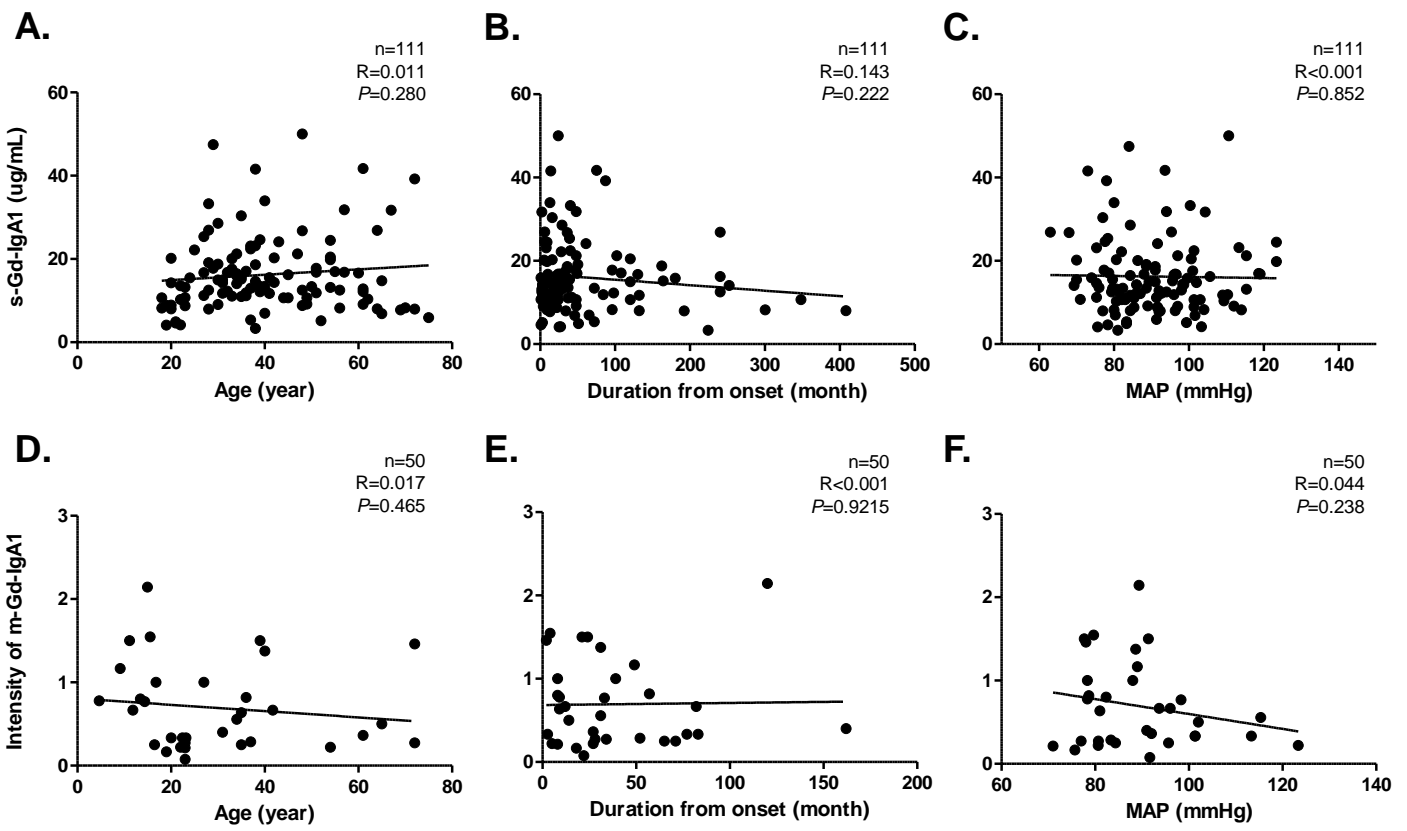

Supplement: S1 Fig — Scatter plots of s-Gd-IgA1 values or m-Gd-IgA1 intensity vs. age (A and D), duration from onset (B and E), and MAP (C and F). Data were statistically analyzed using Spearman correlation tests. (PDF) [file pone.0206865.s001.pdf]

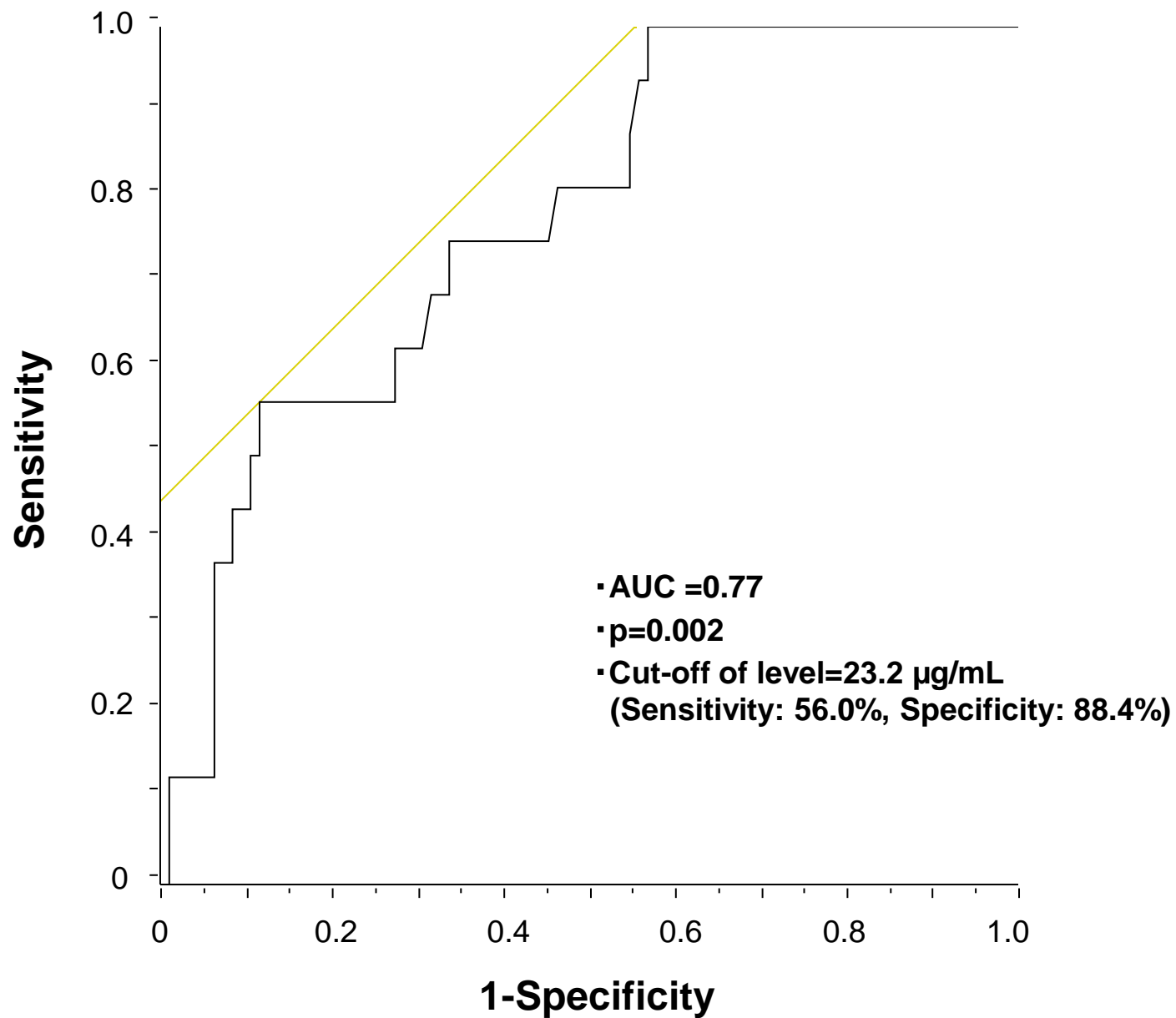

Supplement: S2 Fig — Receiver operator characteristic curves and calculation of AUC for s-Gd-IgA1 value required to predict 30% eGFR reduction in patients with IgAN (n = 111). (PDF) [file pone.0206865.s002.pdf]

HSPN

LN

AAV

MCD

A.

B.

C.

D.

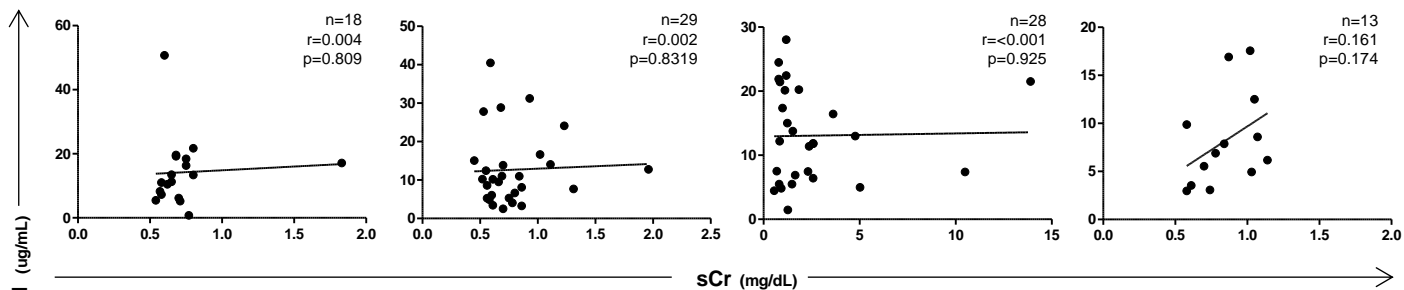

E.

F.

G.

H.

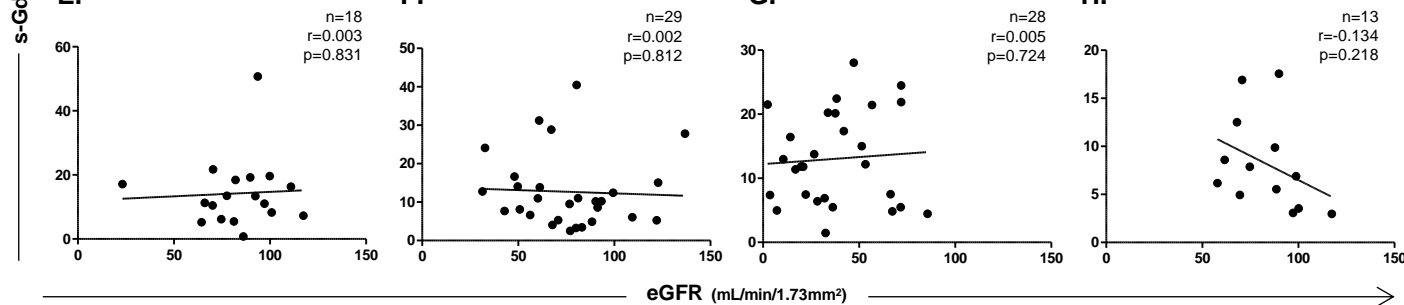

Supplement: S3 Fig — Scatter plots show s-Gd-IgA1 vs. sCr and eGFR in patients with HSPN (A and E), LN (B and F), AAV (C and G), and MCD (D and H). Data were statistically analyzed using Spearman correlation tests. (PDF) [file pone.0206865.s003.pdf]

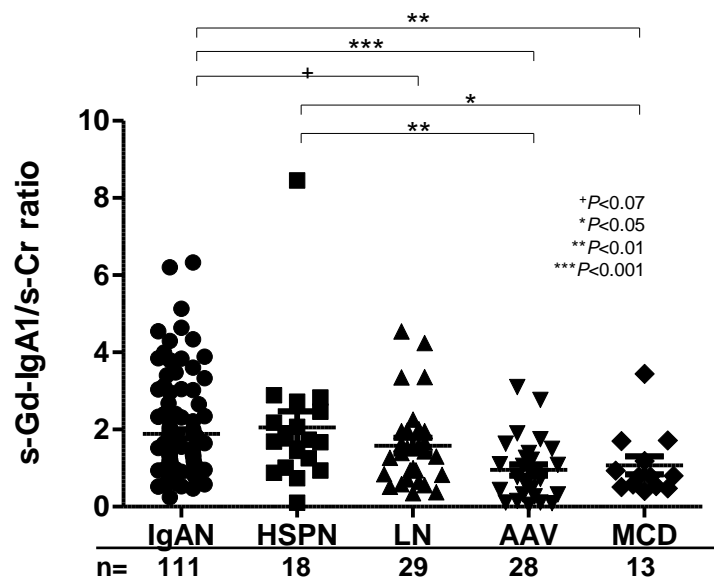

Supplement: S4 Fig — Serum-Gd-IgA1 values divided by sCr values for individual patient were compared among the study groups. Data were statistically analyzed using Mann-Whitney U tests. (PDF) [file pone.0206865.s004.pdf]

IgA deposition

IgG deposition

IgM deposition

A.

B.

C.

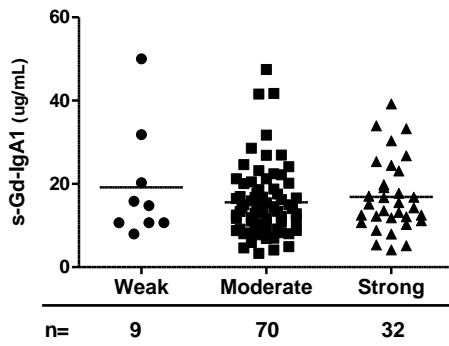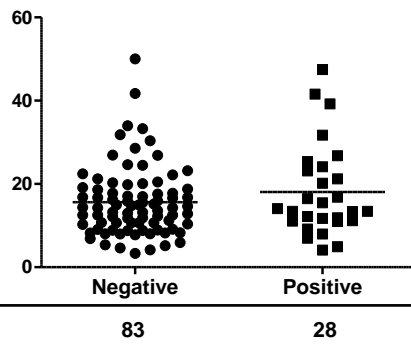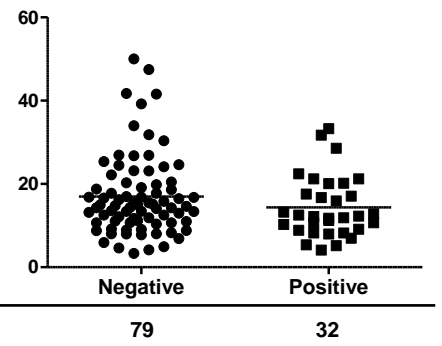

D.

E.

F.

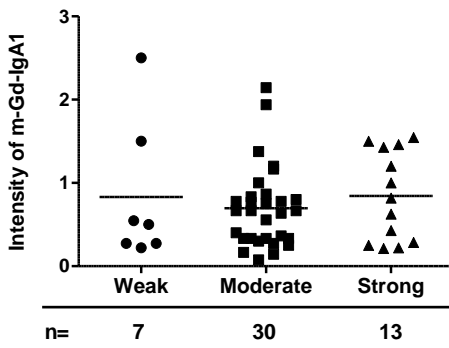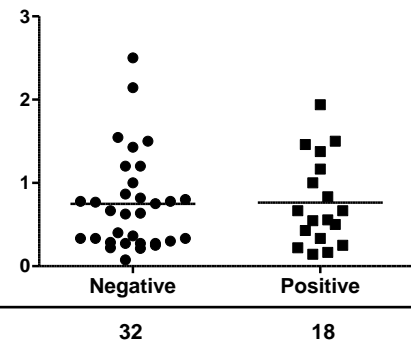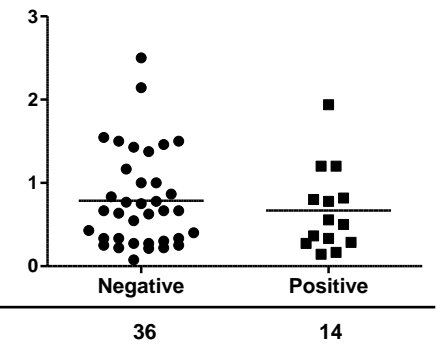

Supplement: S5 Fig — Patients were assigned to groups according to mesangial IgA intensity (A and D), IgG deposition (B and E), and IgM deposition (C and F), then compared with s-Gd-IgA1 values or m-Gd-IgA1 intensity. Horizontal solid lines represent means. Data were statistically analyzed using Kruskal-Wallis tests and Mann-Whitney U tests. (PDF) [file pone.0206865.s005.pdf]
