# Supplementary material for: Clinical significance of serum and mesangial galactose-deficient IgA1 in patients with IgA nephropathy
Source: PLoS One. 2018 Nov 2;13(11):e0206865. doi: 10.1371/journal.pone.0206865 (PMC6214568; doi:10.1371/journal.pone.0206865)
Supplement: S1 Table — (RTF) [file pone.0206865.s006.rtf]

Supplemental Table 1S1�@Table. Risk stratification for dialysis according to the criteria of the Japanese Society of Nephrology [34]34
Clinical Grade	Histological Grade 1	Histological Grade 2	Histological Grade 3 and 4	
1	Low risk	Medium risk	High risk	
2	Medium risk	Medium risk	High risk	
3	High risk	High risk	Super high risk	
