# Supplementary material for: Clinical significance of serum and mesangial galactose-deficient IgA1 in patients with IgA nephropathy
Source: PLoS One. 2018 Nov 2;13(11):e0206865. doi: 10.1371/journal.pone.0206865 (PMC6214568; doi:10.1371/journal.pone.0206865)
Supplement: S2 Table — (RTF) [file pone.0206865.s007.rtf]

Supplemental Table 2S2 Table. Comparison of immunosuppressive treatment among study groups when renal biopsies were obtained


Abbreviations: No (%); number (%), IgAN; Immunoglobulin A nephropathy, HSPN; Henoch-Schönlein purpura nephritis, LN; lupus nephritis, AAV; ANCA-associated vasculitis, MCD; minimal change disease, CyA; cyclosporine A, Tac; tacrolimus, MMF; mycophenolate mofetil. 
